# Determination and Visualization of Peimine and Peiminine Content in *Fritillaria thunbergii* Bulbi Treated by Sulfur Fumigation Using Hyperspectral Imaging with Chemometrics

**DOI:** 10.3390/molecules22091402

**Published:** 2017-08-23

**Authors:** Juan He, Yong He, Chu Zhang

**Affiliations:** 1 Zhejiang Academy of Traditional Chinese Medicine, Key Laboratory of Research and Development of Chinese Medicine of Zhejiang Province, Hangzhou 310007, China; hej0516@126.com; 2 College of Biosystems Engineering and Food Science, Zhejiang University, Hangzhou 310058, China

**Keywords:** near-infrared hyperspectral imaging, *Fritillaria thunbergii* bulbus, peimine, peiminine, prediction map

## Abstract

Rapid, non-destructive, and accurate quantitative determination of the effective components in traditional Chinese medicine (TCM) is required by industries, planters, and regulators. In this study, near-infrared hyperspectral imaging was applied for determining the peimine and peiminine content in *Fritillaria thunbergii* bulbi under sulfur fumigation. Spectral data were extracted from the hyperspectral images. High-performance liquid chromatography (HPLC) was conducted to determine the reference peimine and peiminine content. The successive projection algorithm (SPA), weighted regression coefficient (*Bw*), competitive adaptive reweighted sampling (CARS), and random frog (RF) were used to select optimal wavelengths, while the partial least squares (PLS), least-square support vector machine (LS–SVM) and extreme learning machine (ELM) were used to build regression models. Regression models using the full spectra and optimal wavelengths obtained satisfactory results with the correlation coefficient of calibration (*r_c_*), cross-validation (*r_cv_*) and prediction (*r_p_*) of most models being over 0.8. Prediction maps of peimine and peiminine content in *Fritillaria thunbergii* bulbi were formed by applying regression models to the hyperspectral images. The overall results indicated that hyperspectral imaging combined with regression models and optimal wavelength selection methods were effective in determining peimine and peiminine content in *Fritillaria thunbergii* bulbi, which will help in the development of an online detection system for real-world quality control of *Fritillaria thunbergii* bulbi under sulfur fumigation.

## 1. Introduction

*Fritillaria thunbergii* Miq. (Zhebeimu) is a famous traditional Chinese medicine (TCM) planted in Zhejiang Province, China. The bulbus of *Fritillaria thunbergii* Miq. is used as medicine as it has curative effects in clearing heat, resolving phlegm, relieving cough, and detoxifying [1]. Peimine and Peiminine are major alkaloids in the *Fritillaria thunbergii* bulbi, which play important roles in these curative effects. Determination of peimine and peiminine content in the *Fritillaria thunbergii* bulbi is important for grading, processing, and trading of the *Fritillaria thunbergii* bulbi. Sulfur fumigation (SF) is a widely-used traditional method to prolong traditional Chinese medicine preservation [2]. Although SF may add uncertain side effects to traditional Chinese medicine and is restricted by the Chinese government, it is still widely used due to its relatively low cost. However, it is important to detect chemical components of *Fritillaria thunbergii* bulbi under SF for quality control and sorting.

At present, laboratory-based chemical methods are used to detect peimine and peiminine content in *Fritillaria thunbergii* bulbi, such as high-performance liquid chromatography (HPLC) [3] and gas chromatography-mass spectrometry (GC-MS) [4]. These methods waste considerable amounts of reagents in addition to being expensive, time-consuming, and complex to operate. Development of a rapid and non-destructive method to detect peimine and peiminine content in the *Fritillaria thunbergii* bulbi will be beneficial for the involved industries, planters, and regulators.

Near-infrared spectroscopy is a widely-used, rapid, and non-destructive method to detect quality of traditional Chinese medicine [5,6,7]. However, near-infrared spectroscopy generally collects spectra of traditional Chinese medicines from small sampling points, resulting in the spectra of the entire sampled region not being acquired. However, near-infrared spectroscopy generally acquires information from small spots, and the spectral information of a sample is represented by average spectra of several times of measurements or several small sampling spots.

Hyperspectral imaging is a technique integrating both spectroscopy and imaging techniques. A hyperspectral image is a 3D data cube (x × y × λ), with 2D gray-scale images (x × y) at the spectral wavebands (λ). Hyperspectral imaging has the advantageof acquiring spectral information from the entire sampling area within the hyperspectral images. Hyperspectral imaging acquires spectral information from the entire sample region, the spectral information is more representative than near-infrared spectra acquired from small spots. With the advantage that each pixel within the hyperspectral images has a spectrum, distribution maps to present component content differences within the samples or among different samples can be formed. With distribution maps, visual information of component content and differences within the samples or among the samples will help to achieve on-line real-world application of hyperspectral imaging. Many studies have been reported using the advantage of acquiring representative spectral information from the entire sample region by hyperspectral imaging without forming distribution maps [8,9,10,11], and many studies have also been reported using the advantage of forming distribution maps by hyperspectral imaging [12,13,14,15].

Hyperspectral imaging has been widely studied in various fields, such as agriculture [16], food [17], medicine [18], pharmacy [19], etc. Few studies have been reported the use of hyperspectral imaging for traditional Chinese medicine [20,21]. Furthermore, hyperspectral imaging shows great potential in quality determination of traditional Chinese medicine. 

The objective of this study was to determine peimine and peiminine content in *Fritillaria thunbergii* bulbi treated by sulfur fumigation for quality control using hyperspectral imaging. The specific objectives were: (1) to explore the feasibility of using hyperspectral imaging to determine peimine and peiminine content in *Fritillaria thunbergii* bulbi; (2) to select optimal wavelengths for peimine and peiminine content determination; (3) to form a prediction map of peimine and peiminine content in *Fritillaria thunbergii* bulbi.

## 2. Results and Discussion

### 2.1. Spectral Profiles

**Figure 1:**
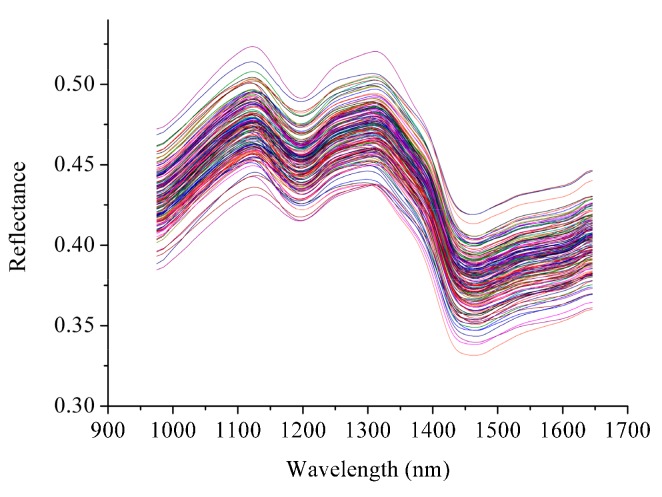
Spectra of *Fritillaria thunbergii* bulbi samples.

Due to the noise in the head and the tail of the spectra, the spectra in the range of 975–1646 nm were used for analysis (Figure 1). All samples showed similar trends with differences found in the reflectance values.

### 2.2. Analysis of Peimine and Peiminine Content under Different Levels of Sulfur Fumigation

**Figure 2:**
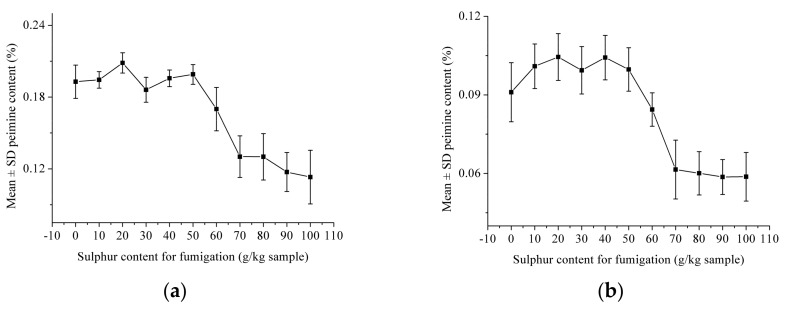
Mean ± standard deviation (SD) for (**a**) peimine and (**b**) peiminine content in *Fritillaria thunbergii* bulbi under different levels of sulfur fumigation.

Peimine and peiminine content (mean ± standard deviation (SD)) in *Fritillaria thunbergii* bulbi under different levels of sulfur fumigation are shown in Figure 2. As shown in Figure 2a, the peimine content showed no significant differences when the sulfur content was 0–50 g/kg. When sulfur content was over 50 g/kg for fumigation, the peimine content decreased with an increase in sulfur content. When the sulfur content was over 70 g/kg, the change of peimine content became quite small. The peiminine content in *Fritillaria thunbergii* bulbi showed a similar trend under different levels of sulfur fumigation.

### 2.3. Sample Set Division

One goal of this study was to build reliable and robust quantitative models to detect peimine and peiminine content in *Fritillaria thunbergii* bulbi. To build these prediction models, the samples were randomly divided into the calibration and prediction sets at the ratio of 3:1, with 124 samples in the calibration set and the remaining 41 samples in the prediction set. No samples were used for both calibration and prediction. The peimine and peiminine content of the prediction set were covered by the calibration set. All the chemometric operations were conducted on the same calibration and prediction set. The statistical analysis of peimine and peiminine content in the calibration and prediction sets is shown in Table 1.

### 2.4. Optimal Wavelength Selection

In hyperspectral images, each pixel contains a spectrum and each wavelength band has a gray-scale image. Hyperspectral images generate a large amount of data. Dealing with such data needs high performance software and hardware. Moreover, the large amount of data also restricts real-world applications of hyperspectral imaging. Spectral data suffer from collinearity and redundancy. Optimal wavelength selection is an efficient way to significantly reduce the amount of data required. Optimal wavelength selection involves choosing a few wavelengths carrying the most useful information for qualitative and quantitative analysis with removal of uninformative wavelengths. In this study, the optimal wavelengths were selected for peimine and peiminine content prediction using the successive projections algorithm (SPA), weighted regression coefficient (*Bw*), competitive adaptive reweighted sampling (CARS), and random frog (RF), which are shown in Table 2. As shown in Table 2, different numbers of wavelengths and different wavelengths were selected by different methods for peimine and peiminine content detection with only minor overlap being observed. Using SPA, *Bw*, CARS, and RF for optimal wavelength selection was based on different principles and different selection criterions, which resulted in different wavelengths being selected. The selected optimal wavelengths were evaluated by performances of calibration models and optimal wavelengths selected by different methods might obtain different prediction results. The objective of using different methods for optimal wavelength selection was to achieve better prediction and simpler models for peimine and peiminine content prediction.

Comparing the number of optimal wavelengths and the number of full spectra wavelengths for peimine detection, the number of wavelengths was significantly reduced from 200 to at most 26, in which is a reduction of at least 87%. The same results were shown for peiminine detection.

### 2.5. Regression Models Using Full Spectra and Optimal Wavelengths

Regression models, including partial least squares (PLS), least-squares support vector machine (LS–SVM), and extreme learning machine (ELM), were built using full spectra (all wavelengths) and optimal wavelengths for the quantitative determination of peimine and peiminine content in *Fritillaria thunbergii* bulbi. Full spectra and optimal wavelengths were used as independent variables ***X*** and reference peimine and peiminine content measured by HPLC were used as dependent variables ***Y*** of the models. The correlation coefficients of calibration (*r_c_*), cross-validation (*r_cv_*) and prediction (*r_p_*) in addition to the root mean square errors of calibration (RMSEC), cross-validation (RMSECV), and prediction (RMSEP) were used for evaluating the model performances. The parameters *r_c_*, *r_cv_*, *r_p_*, RMSEC, RMSECV, and RMSEP were calculated using the predicted peimine and peiminine content and the corresponding reference content measured by HPLC. To build regression models, leave-one-out cross validation was implemented.

For peimine content determination, the results of regression models using full spectra and optimal wavelengths are shown in Table 3. For all regression models, no significant differences were observed beteen *r_c_* and *r_cv_*, indicating the effectiveness of the models. For full spectra models, all models obtained acceptable results, with *r_c_*, and *r_p_* of most models being over 0.8. The ELM model had the best performance with *r_c_* and *r_p_* being over 0.9, while the PLS model obtained the worst results. For PLS models using optimal wavelengths selected by different methods, the SPA–PLS model obtained the best results, while CARS–PLS and *Bw*–PLS models obtained close but slightly worse results. In comparison, the RF–PLS model obtained the worst results with a *r_cv_* of 0.703 and *r_p_* of 0.771. For LS–SVM models using optimal wavelengths selected by different methods, the CARS–LS–SVM model had the best performance, while the SPA–LS–SVM and *Bw*–LS–SVM models obtained close but slightly worse results. Finally, the RF–LS–SVM model obtained worst results with a *r_cv_* of 0.708 and *r_p_* of 0.791. For ELM models using optimal wavelengths selected by different methods, the CARS–ELM model performed best, while SPA–ELM and *Bw*–ELM obtained close but slightly worse results. Finally, the RF–ELM model had the worst performance. In all, for calibration models using optimal wavelengths selected by different methods, ELM models performed the best, while the PLS model obtained the worst results. The CARS–ELM model obtained the best results out of all models using optimal wavelengths.

Comparing the calibration models using optimal wavelengths with models using full spectra, selection by SPA, *Bw* and CARS showed similar results, while calibration models using optimal wavelengths selected by RF showed worse results compared to those using the full spectra. All regression models had *r_cv_* over 0.8, except regression models using optimal wavelengths selected by RF (*r_cv_* lower than 0.8). Considering that the number of wavelengths was reduced by at least 87% after optimal wavelength selection, the selected optimal wavelengths showed good potential in being used for calibration and prediction for peimine content determination.

For determining the peiminine content, the results of regression models using full spectra and optimal wavelengths are shown in Table 4. For all regression models, no significant differences were observed beteen *r_c_* and *r_cv_*, indicating the effectiveness of the models. For full spectra models, all models obtained acceptable results with *r_c_* and *r_p_* of over 0.8. The ELM model performed the best with a *r_c_* of 0.916, *r_cv_* of 0.843, and *r_p_* of 0.872, while the PLS model and LS–SVM model obtained close but slightly worse results. For PLS models using optimal wavelengths selected by different methods, SPA–PLS and *Bw*–PLS obtained relatively better results, while the CARS–PLS and RF–PLS obtained relatively worse results. For LS–SVM models using optimal wavelengths selected by different methods, SPA–LS–SVM and *Bw*–LS–SVM models obtained relatively better results, while the CARS–LS–SVM model had the highest *r_c_*, *r_cv_*, and lowest *r_p_*. For ELM models using optimal wavelengths selected by different methods, SPA–ELM and *Bw*–ELM models obtained relatively better results, while CARS–ELM model had the highest *r_c_* and lowest *r_p_*. In all, for calibration models using optimal wavelengths selected by different methods, ELM models performed the best, while the PLS model obtained the worst results. SPA–ELM and *Bw*–ELM models obtained relatively better results out of all models when using optimal wavelengths. 

Comparing calibration models using optimal wavelengths with models using full spectra, the calibration models using optimal wavelengths selected by SPA and *Bw* showed results that were similar to those using full spectra, while calibration models with optimal wavelengths selected by RF showed worse results. Furthermore, the calibration models using optimal wavelengths selected by CARS showed a similar *r_c_*, *r_cv_*, and lower *r_p_* compared to the corresponding full spectra models. Considering that the number of wavelengths was reduced by at least 87% after optimal wavelength selection, the selected optimal wavelengths showed good potential to be used for calibration and prediction instead of full spectra for peiminine content determination.

For determining peimine and peiminine content, use of optimal wavelengths significantly reduced the number of input wavelengths while maintaining the same performance for the models. As shown in Table 3 and Table 4, the use of selection methods for optimal wavelengths and calibration had a significant influence on the performance in predicting. The results indicated that the selection of optimal wavelengths and regression models was effective for determining peimine and peiminine content. In general, ELM models performed better than LS–SVM models and PLS models with PLS models obtaining relatively worse results. This might be due to LS–SVM and ELM being able to deal with both linear and non-linear data effectively as the spectral data had non-linear information. Calibration models using optimal wavelengths selected by RF obtained worse results than models using other optimal wavelength selection methods, which possibly might be the result of different selection principles. Optimal wavelength selection could help to solve the problem of the large amount of data generated by hyperspectral imaging. On the other hand, the subsequent high cost is a problem influencing the real-world applications of hyperspectral imaging. Optimal wavelength selection would help to select only a few wavelengths to develop low-cost multi-spectral imaging system for real-world applications.

### 2.6. Prediction Maps of Peimine and Peimine Content in Fritillaria thunbergii Bulbi

One of the advantages of hyperspectral imaging is that each pixel within the hyperspectral image contains a spectrum in the spectral range of the system. This allows for prediction of each pixel, which can be used to create the prediction map. The general procedure of forming a prediction map involves first building a robust and representable model, before applying the model to create a pixel-wise spectrum within the hyperspectral images. The prediction value of each pixel was presented as a color.

**Figure 3:**
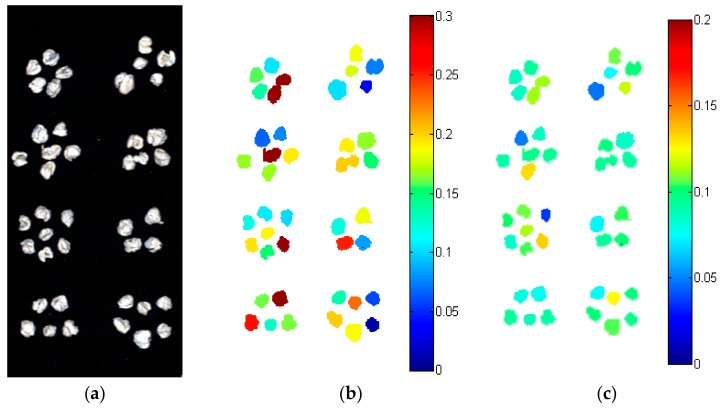
(**a**) Pseudo image of *Fritillaria thunbergii* bulbi (generated from gray-scale images at 1000, 1200, and 1400 nm) in addition to prediction maps of (**b**) peimine and (**c**) peiminine content in *Fritillaria thunbergii* bulbi. The peimine and peiminine content are color-coded.

A hyperspectral image can have thousands or even up to hundreds of thousands of pixels. Thus, the use of full spectra model required intensive computation with a long time period needed. As mentioned above, the optimal wavelength selection could significantly reduce the amount of data and simplify the model. In this study, the regression models using optimal wavelengths were used to form prediction maps for peimine and peiminine content in *Fritillaria thunbergii* bulbi. As shown in Table 3 and Table 4, the CARS–ELM model performed best for predicting peimine content, while the SPA–ELM model performed best for predicting peiminine content. Thus, these two models were used to form prediction maps for peimine and peiminine content in *Fritillaria thunbergii* bulbi. Generally, prediction maps are presented at pixel-wise level with each pixel having a predicted value by using the pixel-wise spectra. High content of components can be observed and collected from the samples for further process based on the prediction maps. However, it was difficult to collect sample regions in *Fritillaria thunbergii* bulbi that corresponded to the pixels with high peimine or peiminine content within the prediction maps. To collect sample regions with high peimine and peiminine content needed precision operations and machines, and it would increase the labor and cost to achieve the goal. For real-world application, a single intact bulbus was used for process under sulfur fumigation. Presenting differences on peimine and peiminine content among different *Fritillaria thunbergii* bulbi provided direct content information for quality control under sulfur fumigation. Knowing the peimine and peiminine content of each bulbus was more effective for quality control and sorting than knowing the pixel-wise value. Thus, the average peimine and peiminine content were predicted for evaluating the quality of *Fritillaria thunbergii* bulbi. The prediction maps of peimine and peiminine content are shown in Figure 3. As shown in Figure 3, different *Fritillaria thunbergii* bulbi showed different peimine and peiminine contents. The predicted maps provided visual information depicting the peimine and peiminine content, which would help for real-world online determination of peimine and peiminine content in *Fritillaria thunbergii* bulbi in addition to quality control and sorting of *Fritillaria thunbergii* bulbi.

## 3. Materials and Methods 

### 3.1. Sample Preparation

Fresh *Fritillaria thunbergii* bulbi were collected from Panan, Zhejiang Province, China. The *Fritillaria thunbergii* bulbi were appraised by pharmacy director of Zhejiang Academy of Traditional Chinese Medicine before further analysis. The fumigation treatments were conducted according to a previous reference [22]. The samples of each treatment were fumigated by 0, 10, 20, 30, 40, 50, 60, 70, 80, 90, and 100 g sulfur per kg sample. For each treatment, 15 samples were prepared with a total of 165 samples being collected. The samples were then used for hyperspectral image acquisition.

### 3.2. Hyperspectral Image Acquisition and Spectra Extraction

Hyperspectral images of the SF-treated *Fritillaria thunbergii* bulbi were acquired using a laboratory-based hyperspectral imaging system. The imaging spectrograph (ImSpector N17E; Spectral Imaging Ltd., Oulu, Finland) coupled with a 320 × 256 camera (Xeva 992; Xenics Infrared Solutions, Leuven, Belgium) was used to acquire hyperspectral images, while an illumination system containing two symmetrically-placed 150 W tungsten halogen lamps (Fiber-Lite DC950 Illuminator; Dolan Jenner Industries Inc., Boxborough, MA, USA) for the imaging spectrograph were used as the light source. The samples were placed on an electric mobile platform (Isuzu Optics Corp., Hsinchu, Taiwan) for line scanning. To acquire non-deformable and clear images, the distance between the samples and the detector, the moving speed of the electric mobile platform and the exposure time of the camera were adjusted to 34.5 cm, 27 mm/s, and 4 ms, respectively.

Following this, the acquired raw hyperspectral images were corrected to reflectance images according to the following equation

(1) $$HSI_{C}=\frac{HSI_{raw}-HSI_{dark}}{HIS_{white}-HSI_{dark}}$$

where $HSI_{C}$ was the corrected image; $HSI_{raw}$ was the raw image; $HIS_{white}$ was the white reference image; and $HSI_{dark}$ was the dark reference image.

To extract spectral information, the sample regions were isolated from the background by applying a mask (binary image) to the corrected images. A grayscale image at 1200 nm was used to form the binary image with a mask, before the mask was applied to remove the background in the grayscale images at each wavelength. After background removal, the pixel-spectrum of each pixel within a sample was preprocessed by wavelet transform (WT) (Daubechies 8 with decomposition level 3) for smoothing to reduce random noises. WT decomposes the spectral data into a high-frequency and low-frequency part. The high frequency part contains noises with soft threshold values being applied to this part to reduce noises. Following this, the low frequency part and the smoothed high frequency part are used to reconstruct the spectra. The average spectrum of each sample is averaged by all pixel-wise spectra within the sample.

### 3.3. Chemical Analysis of Peimine and Peiminine Content in Fritillaria thunbergii Bulbi

After the image acquisition, the *Fritillaria thunbergii* bulbi were powdered for measuring peimine and peiminine content. Peimine and peiminine content were measured by a HPLC machine (LC-20AT, Shimadzu, Kyoto, Japan). First, 2 g of each powdered sample were accurately weighed and put into the flask, while 4 mL of concentrated ammonia solution were added to infiltrate the sample for 1 h. Secondly, a 40-mL mixture of chloroform methanol (4:1) were added, before the solutions were well mixed with the sample. The flask was heated for 2 h over a water bath at 80 °C. Thirdly, after the flask cooled, the mixture of chloroform and methanol (4:1) was added to maintain the same volume before heating. Following this, the mixed solution was filtered, before 10 mL of the filtrate was collected and transferred to an evaporating dish and dried. The residue was dissolved again with methanol in a 2.0 mL calibrated flask.

For HPLC analysis, the chromatographic column used was Capcell Pak C18 (250 × 4.6 mm, 5 μm, Shiseido, Tokyo, Japan), while the mobile phase was comprised of acetonitrile, water, and diethylamine (70:30:0.03). The column temperature and the flow rate was set as 30 °C and 1.00 mL/min, respectively. Evaporative light scattering detector parameters were set as a tube temperature of 100 °C and a carrier gas flow rate of 1.8 L/min. The injection volume was 10 μL and the number of theoretical plates was more than 2000 (calculated by the peak of peimine). The peak area was measured by the above chromatographic conditions. The standard curve of peimine and peiminine was obtained by using the logarithmic value of the standard as X and the peak area as Y. The standard curve of peimine was Y = 1.6154X + 4.9538 (R^2^ = 0.9997), while the standard curve of the peiminine was Y = 1.6234X + 4.7856 (R^2^ = 0.9999). The peimine and peiminine content of the samples were then calculated using the standard curves.

### 3.4. Data Analysis Methods

#### 3.4.1. Regression Methods

Partial least squares (PLS) is a widely-used regression method in spectral data analysis. It decomposes the spectral data matrix (X) and physicochemical properties matrix (Y) simultaneously, before the highest linear relationship between the scores of X and Y are explored. In particular, PLS is a fast and effective method for dealing with a data matrix with more variables than samples, such as spectra data [23].

The least-squares support vector machine (LS–SVM) is a modification of the original support vector machine (SVM). Unlike SVM, LS–SVM uses equality-type constraints instead of inequality-type constraints, before the equality is solved to find solutions for quadratic programming in dual spaces. Similar to SVM, the kernel functions are essential and important for LS–SVM with RBF being a widely-used kernel function for dealing with non-linear issues. The parameters (the kernel width γ and the regularization parameter σ) of LS–SVM should be determined with the grid-search being a widely-used procedure to determine the optimal parameters [24].

The extreme learning machine (ELM) is a fast, simple and effective neural network. In ELM, the number of nodes in the hidden layer and the activation function need to be set. The input weights and the bias of nodes in the hidden layer are randomly generated. The optimal number of nodes in the hidden layer is determined by comparing the performances of ELM models using different numbers of nodes in the hidden layer [25]. 

#### 3.4.2. Optimal Wavelength Selection Methods

Hyperspectral imaging generates a large amount of data and dealing with this large amount of data is challenging. Optimal wavelength selection is an efficient method to reduce the amount of data from hyperspectral images. The optimal wavelength selection aims to select a few wavelengths from the original full spectra, which contributes most for modeling. Uninformative wavelengths are removed. 

Successive projections algorithm (SPA) is a widely-used variable selection method. It projects one wavelength variable onto the others for each iteration. Following this, the wavelengths with maximum projection values are selected as a candidate subset of the optimal wavelengths. A modeling method is then applied to evaluate the performances of the wavelength variables in the subset, before the variables corresponding to the minimum root mean square error (RMSE) are selected [26,27].

Weighted regression coefficient (*Bw*) is a variable selection method based on PLS. *Bw* is acquired by standardizing the spectral data to the same scale during the establishment of PLS model. Since the spectral data are standardized to the same scale, *Bw* can indicate the relative importance of each variable. Variables with higher *Bw* values can be selected as optimal wavelengths [26,27]. 

Competitive adaptive reweighted sampling (CARS) is a variable selection method based on PLS. It maintains a number of variables with higher absolute weights of regression coefficient (RC) by using an exponentially decreasing function (EDF) and adaptive reweighted sampling (ARS) in each iteration. PLS models are then built using the selected variables and the root mean square error of cross validation (RMSECV) is obtained. After N iterations (N is the predefined number of Monte-Carlo sampling), the variable subset corresponding to the PLS model with minimum RMSECVs are selected as the optimal wavelengths [26,28]. 

Random frog (RF) is an efficient approach for variable selection based on PLS, which is a reversible jump Markov Chain that is similar to Monte-Carlo sampling. It starts from an initial subset with the subset being continually updated in each iteration. After N iterations (N is predefined), the selection probability of each variable can be calculated, before the variables with higher selection probability can be selected as optimal wavelengths [26,29].

#### 3.4.3. Model Evaluation and Software

The performances of calibration models were evaluated by the correlation coefficients (*r_c_*, *r_cv_* and *r_p_*) in addition to the root mean square errors of the calibration and prediction sets (RMSEC, RMSECV and RMSEP). The models with higher *r_c_*, *r_cv_* and *r_p_* as well as a lower RMSEC, RMSECV and RMSEP were considered as the better models. PLS and *Bw* were performed on Unscrambler^®^ 10.1 (CAMO AS, Oslo, Norway), while WT, SPA, RF, CARS, ELM, and LS–SVM were performed on MATLAB R 2010b (The Math Works, Natick, MA, USA).

## 4. Conclusions

In this study, we proposed a rapid and non-destructive method for determination of peimine and peiminine content in *Fritillaria thunbergii* bulbi by hyperspectral imaging. Hyperspectral images covering the spectral range of 874–1734 nm were acquired. Spectral data were acquired from the hyperspectral images. Regression models by PLS, LS–SVM, and ELM were built using full spectral and the optimal wavelengths selected by SPA, *Bw*, CARS, and RF. The satisfactory results of these models indicated the effectiveness of optimal wavelength selection methods and regression models in determining peimine and peiminine content. For regression models, the ELM models using full spectra and optimal wavelengths performed better than corresponding LS–SVM models and PLS models. For optimal wavelength selection methods, the regression models using optimal wavelengths selected by RF performed worse compared to models using selection methods of *Bw*, SPA, and CARS. Based on the features of hyperspectral imaging, the prediction maps of peimine and peiminine content in *Fritillaria thunbergii* bulbi were obtained with high accuracy and efficient performance. The results indicated the effectiveness of optimal wavelength selection and regression methods in determining the peimine and peiminine content by hyperspectral imaging combined with chemometrics. Hyperspectral imaging had the potential to be used in the quality control and sorting of *Fritillaria thunbergii* bulbi and other TCMs under sulfur fumigation, which can help the development of an online detection system for real-world applications.

## Figures and Tables

**Table 1:** Statistical analysis of peimine and peiminine content in the calibration and prediction sets.

	Calibration Set			Prediction Set		
	Range (%)	Mean (%)	SD (%)	Range (%)	Mean (%)	SD (%)
Peimine	0.0729–0.2261	0.1678	0.0387	0.1025–0.2119	0.1647	0.0353
Peiminine	0.0382–0.1203	0.0849	0.0212	0.0422–0.1120	0.0811	0.0203

**Table 2:** Optimal wavelengths selected for peimine and peiminine content prediction by SPA, *Bw*, CARS, and RF.

	Methods ^a^	Number	Wavelength (nm)
Peimine	SPA	9	1558, 1517, 1416, 1372, 1646, 1035, 999, 1456, 1234
	*Bw*	13	975, 1042, 1123, 1207, 1291, 1338, 1372, 1413, 1456, 1483, 1558, 1609, 1646
	CARS	26	978, 988, 999, 1002, 1009, 1015, 1025, 1035, 1039, 1049, 1066, 1220, 1234, 1241, 1274, 1389, 1396, 1419, 1440, 1477, 1494, 1497, 1514, 1517, 1544, 1639
	RF	26	1521, 1517, 1039, 1544, 1497, 1500, 1558, 1035, 1009, 1015, 1244, 995, 1002, 1234, 1210, 1059, 1241, 1494, 1019, 1561, 1062, 1551, 988, 999, 1207, 1514
Peiminine	SPA	8	1379, 1348, 999, 1305, 975, 1416, 1646, 1544
	*Bw*	13	975, 1012, 1126, 1164, 1244, 1335, 1375, 1423, 1460, 1490, 1558, 1609, 1646
	CARS	21	1005, 1019, 1042, 1059, 1082, 1210, 1230, 1244, 1332, 1345, 1365, 1369, 1514, 1521, 1534, 1554, 1558, 1575, 1592, 1598, 1619
	RF	26	1019, 1521, 1578, 1595, 1592, 1575, 1554, 1619, 1517, 1005, 1615, 1598, 1558, 1544, 1244, 1588, 1234, 1524, 1015, 1342, 1500, 1247, 995, 1345, 999, 1039

^a^ In the methods, SPA refers to successive projections algorithm; *Bw* refers to weighted regression coefficients; RF refers to random frog; and CARS refers to competitive adaptive reweighted sampling.

**Table 3:** Results of regression models for peimine content determination.

	Models	Parameters ^a^	Calibration Set				Prediction Set	
			*r_c_*	RMSEC (%)	*r_cv_*	RMSECV (%)	*r_p_*	RMSEP (%)
Full spectra	PLS	7	0.868	0.0192	0.843	0.0208	0.853	0.0210
	LS–SVM	2.0059 × 10^10^ 1.8416 × 10^9^	0.890	0.0176	0.849	0.0204	0.863	0.0204
	ELM	33	0.907	0.0163	0.839	0.211	0.905	0.0200
SPA ^b^	PLS	7	0.876	0.0186	0.851	0.0202	0.875	0.0192
	LS–SVM	1.7088 × 10^9^ 8.4531 × 10^6^	0.880	0.0183	0.855	0.0200	0.867	0.0196
	ELM	35	0.911	0.0159	0.835	0.0221	0.886	0.0198
*Bw* ^b^	PLS	7	0.871	0.0189	0.849	0.0204	0.861	0.0201
	LS–SVM	3.113 × 10^8^ 6.5634 × 10^6^	0.881	0.0182	0.853	0.0201	0.856	0.0203
	ELM	34	0.907	0.0163	0.852	0.0205	0.890	0.0196
CARS ^b^	PLS	9	0.879	0.0183	0.842	0.0208	0.860	0.0210
	LS–SVM	1.3383 × 10^11^ 2.0197 × 10^7^	0.909	0.0160	0.860	0.0197	0.883	0.0208
	ELM	36	0.918	0.0153	0.858	0.0199	0.898	0.0224
RF ^b^	PLS	12	0.802	0.0230	0.703	0.0276	0.771	0.0270
	LS–SVM	3.0511 × 10^10^ 2.6138 × 10^6^	0.826	0.0218	0.708	0.0273	0.791	0.0260
	ELM	39	0.844	0.0206	0.720	0.0271	0.818	0.0270

^a^ parameters means the parameters of the regression models of each dataset. For PLS model, parameter is the optimal number of latent variables (LVs); for LS–SVM model, parameter is the kernel width γ and the regularization parameter σ^2^; and for ELM model, parameter is the number of nodes in the hidden layer. ^b^ SPA refers to successive projections algorithm; *Bw* refers to weighted regression coefficients; RF refers to random frog; and CARS refers to competitive adaptive reweighted sampling.

**Table 4:** Results of regression models for peiminine content determination.

	Models	Parameters ^a^	Calibration Set				Prediction Set	
			*r_c_*	RMSEC (%)	*r_cv_*	RMSECV (%)	*r_p_*	RMSEP (%)
Full spectra	PLS	8	0.867	0.0105	0.832	0.0117	0.853	0.0115
	LS–SVM	2.2493 × 10^9^ 3.1678 × 10^7^	0.908	0.0089	0.848	0.0112	0.850	0.0123
	ELM	34	0.916	0.0085	0.843	0.0114	0.872	0.0120
SPA ^b^	PLS	7	0.874	0.0102	0.855	0.0109	0.846	0.0119
	LS–SVM	9.5254 × 10^9^ 7.8625 × 10^7^	0.875	0.0102	0.855	0.0109	0.846	0.0119
	ELM	35	0.901	0.0092	0.842	0.0114	0.852	0.0127
*Bw* ^b^	PLS	7	0.865	0.0106	0.841	0.0114	0.865	0.0109
	LS–SVM	4.7160 × 10^10^ 9.2121 × 10^8^	0.877	0.0101	0.848	0.0112	0.855	0.0114
	ELM	15	0.878	0.0101	0.860	0.0108	0.867	0.0111
CARS ^b^	PLS	10	0.888	0.0097	0.853	0.0110	0.824	0.0131
	LS–SVM	1.3132 × 10^11^ 2.4395 × 10^7^	0.911	0.0087	0.869	0.0104	0.807	0.0141
	ELM	25	0.907	0.0089	0.871	0.0104	0.816	0.0174
RF ^b^	PLS	8	0.853	0.0110	0.819	0.0121	0.823	0.0125
	LS–SVM	1.9460 × 10^10^ 1.8240 × 10^7^	0.868	0.0105	0.822	0.0120	0.831	0.0124
	ELM	31	0.885	0.0098	0.823	0.0122	0.830	0.0135

^a^ parameters means the parameters of the regression models of each dataset. For PLS model, parameter is the optimal number of latent variables (LVs); for LS–SVM model, parameter is the kernel width γ and the regularization parameter σ^2^; and for ELM model, parameter is the number of nodes in the hidden layer. ^b^ SPA refers to successive projections algorithm; *Bw* refers to weighted regression coefficients; RF refers to random frog; and CARS refers to competitive adaptive reweighted sampling.

## References

[1] Duan B., Huang L., Chen S. (2012). Study on the destructive effect to inherent quality of *Fritillaria thunbergii* Miq. (Zhebeimu) by sulfur-fumigated process using chromatographic fingerprinting analysis. Phytomed. Int. J. Phytother. Phytopharm..

[2] Jiang X., Huang L.F., Zheng S.H., Chen S.L. (2013). Sulfur fumigation, a better or worse choice in preservation of Traditional Chinese Medicine?. Phytomed. Int. J. Phytother. Phytopharm..

[3] Li S.L., Lin G., Chan S.W., Li P. (2001). Determination of the major isosteroidal alkaloids in bulbs of Fritillaria by high-performance liquid chromatography coupled with evaporative light scattering detection. J. Chromatogr. A.

[4] Li S.L., Chan S.W., Li P., Lin G., Zhou G.H., Ren Y.J., Chiu F.C. (1999). Pre-column derivatization and gas chromatographic determination of alkaloids in bulbs of Fritillaria. J. Chromatogr. A.

[5] Chan C.O., Chu C.C., Mok K.W., Chau F.T. (2007). Analysis of berberine and total alkaloid content in Cortex Phellodendri by near infrared spectroscopy (NIRS) compared with high-performance liquid chromatography coupled with ultra-visible spectrometric detection. Anal. Chim. Acta.

[6] Nie L., Dai Z., Ma S. (2016). Enhanced accuracy of near-infrared spectroscopy for traditional Chinese medicine with competitive adaptive reweighted sampling. Anal. Lett..

[7] Wang X., Wang X., Guo Y. (2017). Rapidly simultaneous determination of six effective components in cistanche tubulosa by near infrared spectroscopy. Molecules.

[8] Erkinbaev C., Henderson K., Paliwal J. (2017). Discrimination of gluten-free oats from contaminants using near infrared hyperspectral imaging technique. Food Control.

[9] Zhang N., Liu X., Jin X. (2017). Determination of total iron-reactive phenolics, anthocyanins and tannins in wine grapes of skins and seeds based on near-infrared hyperspectral imaging. Food Chem..

[10] Washburn K.E., Stormo S.K., Skjelvareid M.H., Heia K. (2017). Non-invasive assessment of packaged cod freeze-thaw history by hyperspectral imaging. J. Food Eng..

[11] Munera S., Besada C., Aleixos N., Talens P., Salvador A., Sun D-W., Cubero S., Blasco J. (2017). Non-destructive assessment of the internal quality of intact persimmon using colour and VIS/NIR hyperspectral imaging. LWT-Food Sci. Technol..

[12] Zheng X., Peng Y., Wang W. (2017). A nondestructive real-time detection method of total viable count in pork by hyperspectral imaging technique. Appl. Sci..

[13] Mollazade K. (2017). Non-destructive identifying level of browning development in button mushroom (agaricus bisporus) using hyperspectral imaging associated with chemometrics. Food Anal. Methods.

[14] Mo C., Kim M.S., Kim G., Lim J., Delwiche S.R., Chao K., Lee H., Cho B.K. (2017). Spatial assessment of soluble solid contents on apple slices using hyperspectral imaging. Biosyst. Eng..

[15] Shi J.Y., Hu X.T., Zou X.B., Zhao J.W., Zhang W., Holmes M., Huang X.W., Zhu Y.D., Li Z.H., Shen T.T. (2017). A rapid and nondestructive method to determine the distribution map of protein, carbohydrate and sialic acid on Edible bird’s nest by hyper-spectral imaging and chemometrics. Food Chem..

[16] Bock C.H., Poole G.H., Parker P.E., Gottwald T.R. (2010). Plant disease severity estimated visually, by digital photography and image analysis, and by hyperspectral imaging. Crit. Rev. Plant Sci..

[17] Gowen A.A., O'Donnell C.P., Cullen P.J., Downey. G., Frias J.M. (2007). Hyperspectral imaging—An emerging process analytical tool for food quality and safety control. Trends Food Sci. Technol..

[18] Manea D. (2014). Hyperspectral imaging in the medical field: Present and future. Appl. Spectrosc. Rev..

[19] Gowen A.A., O‘Donnell C.P., Cullen P.J., Bell S.E. (2008). Recent applications of Chemical Imaging to pharmaceutical process monitoring and quality control. Eur. J. Pharm. Biopharm..

[20] Zhang H., Wu T., Zhang L., Zhang P. (2016). Development of a portable field imaging spectrometer: Application for the identification of sun-dried and sulfur-fumigated Chinese herbals. Appl. Spectrosc..

[21] Sandasi M., Vermaak I., Chen W., Viljoen A.M. (2014). Hyperspectral imaging and chemometric modeling of echinacea—a novel approach in the quality control of herbal medicines. Molecules.

[22] He J., Zhang C., He Y. (2017). Application of near-infrared hyperspectral imaging to detect sulfur dioxide residual in the *Fritillaria thunbergii* bulbus treated by sulfur fumigation. Appl. Sci..

[23] Geladi P., Kowalski B.R. (1986). Partial least-squares regression: A tutorial. Anal. Chim. Acta.

[24] Zhang C., Xu N., Luo L., Liu F., Kong W., Feng L., He Y. (2014). Detection of aspartic acid in fermented cordyceps powder using near infrared spectroscopy based on variable selection algorithms and multivariate calibration methods. Food Bioprocess Technol..

[25] Huang G.B., Zhu Q.Y., Siew C.K. (2006). Extreme learning machine: Theory and applications. Neurocomputing.

[26] Zhang C., Jiang H., Liu F., He Y. (2016). Application of near-infrared hyperspectral imaging with variable selection methods to determine and visualize caffeine content of coffee beans. Food Bioprocess Technol..

[27] Zhang C., Liu F., Kong W., He Y. (2015). Application of visible and near-infrared hyperspectral imaging to determine soluble protein content in oilseed rape leaves. Sensors.

[28] Li H., Liang Y., Xu Q., Cao D. (2009). Key wavelengths screening using competitive adaptive reweighted sampling method for multivariate calibration. Anal. Chim. Acta.

[29] Li H.D., Xu Q.S., Liang Y.Z. (2012). Random frog: An efficient reversible jump Markov chain Monte Carlo-like approach for variable selection with applications to gene selection and disease classification. Anal. Chim. Acta.

